# Characterizing patients who underwent ovarian tissue cryopreservation at a large academic center in the United States

**DOI:** 10.1016/j.xfre.2025.09.006

**Published:** 2025-10-01

**Authors:** Meridith P. Pollie, Caitlin E. Martin, Margaret A. Rush, Claire Carlson, Jeanne Ricci, Maddie Trego, Peter Mattei, Suneeta Senapati, Jill P. Ginsberg, Clarisa R. Gracia

**Affiliations:** a Department of Obstetrics and Gynecology, University of Pennsylvania, Philadelphia, Pennsylvania; b Division of Reproductive Endocrinology and Infertility, Department of Obstetrics and Gynecology, University of Pennsylvania, Philadelphia, Pennsylvania; c Division of Oncology, The Children’s Hospital of Philadelphia, Philadelphia, Pennsylvania; d Division of General, Thoracic and Fetal Surgery at Children's Hospital of Philadelphia, The Children’s Hospital of Philadelphia, Philadelphia, Pennsylvania

**Keywords:** Ovarian tissue cryopreservation, fertility preservation, oncofertility

## Abstract

**Objective:**

To describe characteristics and outcomes of ovarian tissue cryopreservation (OTC) patients over nearly 2 decades of institutional experience.

**Design:**

Retrospective observational study.

**Subjects:**

All patients who underwent OTC for any indication from January 1, 2008 to November 1, 2024.

**Exposure:**

Ovarian tissue cryopreservation at a combined academic fertility program at the Children’s Hospital of Philadelphia and the Hospital of the University of Pennsylvania.

**Main Outcome Measures:**

Fertility outcomes were assessed, including oocyte and embryo vitrification, autologous ovarian tissue transplantation (OTT), pregnancies, and live births. Additionally, gonadotoxic treatment exposure and association with post-OTC outcomes, such as the development of premature ovarian insufficiency, hormone replacement initiation, and overall survival, were evaluated.

**Results:**

Of 216 patients who underwent OTC, 39 had procedures at Hospital of the University of Pennsylvania (median age, 24.4 ± 7.8 years), and 177 underwent procedures at the Children’s Hospital of Philadelphia (10.3 ± 5.9 years). Malignancy was the indication for 77.8% of OTC procedures, whereas 22.2% of procedures had benign indications. Ovarian tissue cryopreservation procedures (biopsy, 92.1%; oophorectomy, 7.9%) were performed laparoscopically in most patients, with a small minority of procedures performed abdominally as part of a concurrent open procedure for clinical care. Concomitant oophoropexy was performed in 16 patients (7.4%) at the time of OTC. Additional fertility procedures included oocyte vitrification (n = 35, 25.7% via stimulation and 74.3% from the ovarian tissue) and embryo vitrification (n = 1, 0.5%). One patient underwent OTT but ultimately needed donor oocytes to have a live birth. Five patients had unassisted pregnancies with live births, and 2 patients had a live birth after an intrauterine insemination. Of the 208 patients (96.7%) who received chemotherapy after OTC, 198 (95.2%) were exposed to alkylating agents. Of 115 patients with follow-up data, 68.8% were diagnosed with premature ovarian insufficiency, and 63.6% initiated hormone replacement. At time of analysis, 29 patients (13.4%) were deceased.

**Conclusion:**

Ovarian tissue cryopreservation minimizes treatment delay and offers autologous fertility preservation. Although mortality rates in our cohort are relatively high, OTC provides the opportunity to preserve fertility, and some conceive without assistance. At our institution, only one patient has returned to undergo OTT. Additional studies are needed to understand patient factors that may contribute to tissue utilization to improve counseling and increase transplantation rates.

Ovarian tissue cryopreservation (OTC) is a fertility preservation option for patients at risk of premature ovarian insufficiency (POI) because of disease or gonadotoxic treatment. Genetic conditions such as Turner syndrome and galactosemia often cause ovarian dysfunction. Similarly, chemotherapy, radiotherapy, and ovarian surgery can result in POI and infertility. In OTC, autologous tissue is preserved before disease-associated POI or exposure to gonadotoxic treatment.

Although still emerging in the United States, OTC has been used for decades and is standard of care in many European countries (1). Ovarian tissue cryopreservation offers several advantages over oocyte and embryo cryopreservation: prepubertal patients can undergo OTC without ovarian stimulation; it can be performed promptly without delaying cancer therapy; and it preserves a larger pool of potential oocytes, compared with the mean expected yield of oocytes of 10–15 from oocyte retrieval (2, 3). In addition, OTC can offer the potential for multiple transplants if sufficient tissue is removed and provides the potential for autologous hormone replacement and natural conceptions without the need for in vitro fertilization (4, 5). Ovarian tissue transplantation (OTT) after OTC has resulted in over 200 live births, with more reported births every year.

Despite these benefits, rates of OTT remain low (from 3.7% to 8.7%) (6, 7, 8). This study aimed to leverage our experience with OTC in pediatric and adult patients to characterize patient and procedural factors, as well as outcomes related to subsequent OTT, fertility, and cancer-related treatment and mortality.

## Materials and methods

### Study population and design

An experimental OTC protocol was developed at the Hospital of the University of Pennsylvania (HUP) in collaboration with the Oncofertility Consortium and the National Physicians Cooperative. Institutional Review Board approval was obtained at HUP and the Children’s Hospital of Philadelphia (CHOP). This retrospective observational study included patients who underwent OTC at these institutions from 2008 to 2024.

At HUP, participants were 18–42 years old with both ovaries and at risk of ovarian failure because of imminent medical or surgical treatment or an underlying condition. Exclusion criteria included a high risk of surgical complications, serum follicle-stimulating hormone level of >20 mlU/mL without recent chemotherapy exposure, known breast cancer susceptibility gene mutation, suspected ovarian malignancy, and/or the presence of large ovarian masses. Eligible subjects were offered oophorectomy or ovarian biopsy. Beginning in 2015, HUP began offering OTC as a clinical protocol, rather than a research protocol, allowing anyone with a clinical indication to undergo OTC at the discretion of their provider.

At CHOP, because of concerns about the vulnerability of children in relation to the risk/benefit ratio of the proposed procedure, the Institutional Review Board initially restricted OTC eligibility to females aged ≥10 years and allowed for ovarian biopsy only but was later expanded to include patients ≥1-year-old and allow for oophorectomy. Eligible subjects were limited to those at highest risk of long-term ovarian dysfunction because of underlying genetic conditions or exposure to alkylating agents, radiation with potential impact on the ovaries, or stem cell transplant conditioning. The protocol initially allowed for OTC to be performed as a stand-alone surgery but was later amended to require a concurrent clinical procedure to minimize anesthesia risks. Previous therapy was not an exclusion. Children’s Hospital of Philadelphia continues to offer OTC only within a research protocol, allowing for ovarian tissue collection for potential future clinical use and tracking of longitudinal OTC patient outcomes, as well as the development of a tissue bank to advance the science of in vitro maturation of oocytes from cryopreserved ovarian tissue. A timeline highlighting key institutional and clinical milestones related to OTC implementation is shown in Supplemental Figure 1 (available online).

### OTC protocols

Ovarian tissue was cryopreserved using modifications of the techniques described by Gosden et al. (9) and procedures specific to our institution (previously described) (10). For prepubescent patients, the cortical biopsy usually consisted of removing one third to one half of the ovary, whereas in postpubescent patients, approximately one fourth to one third of the ovary was removed. Removed tissue was sliced into strips for cryopreservation measuring approximately 2 cm × 0.5 cm. Originally, in addition to cortical strip cryopreservation, mature or immature oocytes recovered from the ovarian tissue/media were vitrified for all patients; later, the protocol was modified so that oocytes were vitrified only in patients aged ≥10 years. In the original protocol, all oocytes, including immature metaphase I (MI) oocytes and germinal vesicles (GVs), were vitrified on the day of tissue collection. In 2020, the practice evolved so that immature oocytes were cultured for ≤48 hours and only metaphase II (MII) oocytes were vitrified. After cryopreservation, ovarian tissue was stored in cryovials or straws in liquid nitrogen tanks.

### Data collection

Electronic medical records were reviewed to collect demographics, oncologic treatment information, and cancer- and pregnancy-related outcomes. Demographics included age, self-reported race/ethnicity, and age at menarche for postpubertal patients. Ovarian tissue cryopreservation–related variables included indication, surgical route and type, and concurrent fertility-related procedures (e.g., oophoropexy). Fertility-related outcomes included additional fertility procedures (e.g., oocyte or embryo vitrification), number and stage of oocytes retrieved and cryopreserved, posttreatment POI diagnosis, hormone replacement therapy (HRT) use, resumption of menses, follow-up with the fertility team, subsequent OTT, pregnancy, and live birth. Cancer-related outcomes included chemotherapy, radiation exposure, and death.

## Results

A total of 216 patients underwent OTC: 39 patients at HUP (median age, 24.4 ± 7.8 years) and 177 patients at CHOP (mean age, 10.3 ± 5.9 years) (Table 1). Most identified as White/non-Hispanic (HUP, 74.4%; CHOP, 58.8%). Almost all HUP patients (92.1%; age at menarche, 12.4 ± 1.5 years) and one third of CHOP patients (38.4%; age at menarche, 12.2 ± 1.6 years) had reached menarche at the time of OTC. There were 22 HUP patients (56.4%) and 124 CHOP patients (70.1%) who received chemotherapy before OTC, of whom 62.3% received an alkylating agent.

**Table 1:** Patient and procedure characteristics.

Patient/procedure characteristic	Total (n = 216)	HUP (n = 39)	CHOP (n = 177)
Age (y) at OTC	12.8 ± 8.3	24.4 ± 7.8	10.3 ± 5.9
Race			
White/non-Hispanic	133 (61.6%)	29 (74.4%)	104 (58.8%)
Black/non-Hispanic	33 (15.3%)	3 (7.7%)	30 (16.9%)
Asian/non-Hispanic	17 (7.9%)	2 (5.1%)	15 (8.5%)
Hispanic	10 (4.6%)	1 (2.6%)	9 (5.1%)
Other	19 (8.8%)	2 (5.1%)	17 (9.6%)
Not specified	4 (1.9%)	2 (5.1%)	2 (1.1%)
Menarche before OTC	103 (47.7%)	35 (92.1%)	68 (38.4%)
Age at menarche (y)	12.3 ± 1.6	12.4 ± 1.5	12.2 ± 1.6
Pre-OTC chemotherapy	146 (67.6%)	22 (56.4%)	124 (70.1%)
Pre-OTC alkylating agent	91 (62.3%)	11 (50%)	80 (64.5%)
Procedure diagnosis			
Benign	48 (22.2%)	2 (5.1%)	46 (26.0%)
Turner syndrome	2 (0.9%)	2 (5.1%)	0 (0%)
Thalassemia	11 (5.1%)	0 (0%)	11 (6.2%)
Aplastic anemia	11 (5.1%)	0 (0%)	11 (6.2%)
Sickle cell disease	11 (5.1%)	0 (0%)	11 (6.2%)
Other benign	13 (6.0%)	0 (0%)	13 (7.3%)
Malignant	168 (77.8%)	37 (94.9%)	131 (74.0%)
Leukemia	69 (31.9%)	7 (17.9%)	62 (35.0%)
Lymphoma	30 (13.9%)	15 (38.5%)	15 (8.5%)
Breast	5 (2.3%)	5 (12.8%)	0 (0%)
Sarcoma	25 (11.6%)	3 (7.7%)	22 (12.4%)
Other malignant	39 (18.1%)	7 (17.9%)	32 (18.1%)
Procedure type			
Biopsy	199 (92.1%)	22 (56.4%)	177 (100%)
Unilateral	198 (91.7%)	21 (53.8%)	177 (100%)
Bilateral	1 (81.9%)	1 (2.6%)	0 (0%)
Oophorectomy	17 (7.9%)	17 (43.6%)	0 (0%)
Unilateral	16 (7.4%)	16 (41.0%)	0 (0%)
Bilateral	1 (0.5%)	1 (2.6%)	0 (0%)
Concurrent oophoropexy	16 (7.4%)	9 (23.1%)	7 (4.0%)

*Note:* Data are means ± standard deviations or n (%). CHOP = Children’s Hospital of Philadelphia; HUP = Hospital of the University of Pennsylvania; OTC = ovarian tissue cryopreservation.

At HUP, almost all patients had a malignancy (total, 94.9%; lymphoma, 38.5%; leukemia, 17.9%; breast, 12.8%; cervix, 10.3%; sarcoma, 7.7%; colorectal, 5.1%; brain, 2.6%), and two patients (5.1%) underwent OTC for Turner syndrome. At CHOP, 74.0% of patients underwent OTC for malignancy (leukemia, 35.0%; sarcoma, 12.4% [rhabdomyosarcoma, 7.3%; Ewing sarcoma, 3.4%; other sarcoma, 1.7%); neuroblastoma, 9.0%; lymphoma, 8.5%; Wilms tumor, 2.8%; brain tumor, 2.8%; other malignant conditions, 2.8%). Benign conditions were the indication for 26.0% of procedures at CHOP (thalassemia, 6.2%; aplastic anemia, 6.2%; sickle cell disease, 6.2%; primary immunodeficiency, 4.0%; other benign conditions, 3.4%).

Ovarian tissue cryopreservation was performed laparoscopically in all HUP patients and most CHOP patients; some CHOP patients had an abdominal approach as part of a concurrent open procedure (i.e., tumor resection). No intraoperative complications occurred. At CHOP, all OTC procedures were unilateral ovarian biopsies. At HUP, 16 (41.0%) were unilateral oophorectomies, 1 (2.6%) was a bilateral oophorectomy, 21 (53.8%) were unilateral ovarian biopsies, and 1 (2.6%) was a bilateral biopsy. The patient who underwent bilateral oophorectomy had a history of metastatic estrogen receptor–positive breast cancer; therefore, surgical removal of both ovaries was recommended as part of her oncologic care. Although OTT would not be recommended for this patient, she wished to pursue OTC in the event that future research made it possible to mature oocytes from tissue for in vitro fertilization. The mean numbers of strips of ovarian tissue frozen were 7 (range, 1–33) in patients who had an ovarian biopsy and 22 (range, 2–50) in patients who had a whole ovary removed. All tissue was cryopreserved using the slow freeze protocol. At CHOP, 173 (97.7%) of OTC was performed concurrently with another procedure, such as tumor resection, bone marrow aspiration, or central line placement. Concomitant oophoropexy was performed in 16 patients (7.4%).

There were two postoperative complications. One pediatric patient with leukemia experienced an intra-abdominal bleed after ovarian biopsy requiring return to the operating room. She presented postoperative day 19 with abdominal pain, ascites, and a decrease in the hemoglobin level in the setting of thrombocytopenia because of bone marrow transplant conditioning. She was treated for presumed hepatic sinusoidal obstructive syndrome with defibrotide; however, her condition worsened. Ultrasound imaging 1 week later demonstrated echogenic ascites with increasing abdominal girth, and a paracentesis evacuated frank blood. On postoperative day 29, she underwent a laparoscopic evacuation of hemoperitoneum and hemostasis of bleeding from the right ovarian remnant. The second patient, a 25-year-old with leukemia, developed a localized vesicular rash at the suprapubic port site after unilateral oophorectomy consistent with contact dermatitis, attributed to topical bacitracin, which resolved after discontinuation of the medication.

Table 2 presents reproductive and oncologic outcomes. Additional fertility procedures were performed in both HUP and CHOP patients and included oocyte vitrification (n = 35, 16.2%) and embryo vitrification (n = 1, 0.5%). A total of nine patients (age, 23.7 ± 5.3 years) in our cohort froze oocytes after undergoing ovarian stimulation and retrieval. Characteristics of these nine patients are shown in Table 3. The protocols used for stimulation were random start antagonist protocols with the dose depending on ovarian reserve assessment (antral follicle count and/or antimüllerian [AMH]). Six of the nine patients who went through ovarian stimulation and retrieval were exposed to chemotherapy before ovarian stimulation, and three of the six were exposed to alkylating agents. Two of the nine patients had diminished ovarian reserve (AMH levels, 0.3 and 0.4 ng/mL, respectively) documented before stimulation, whereas three of the nine patients did not have this information available clinically. Among the nine patients at our institution who underwent ovarian stimulation for oocyte cryopreservation before subsequent OTC, four underwent OTC within 2–6 days after oocyte retrieval, 2 underwent OTC at 14 and 19 days after retrieval, one patient (with beta thalassemia) underwent OTC approximately six months later, and two underwent stimulation at outside institutions for which interval data were not available. Seven HUP patients froze oocytes via ovarian stimulation before OTC (median oocytes retrieved, 6 [interquartile range {IQR}, 5–10; 100% with MII oocytes), and two CHOP patients froze oocytes via ovarian stimulation (2–4 oocytes retrieved, 100% MII oocytes). One HUP patient froze three embryos at the 2-pronuclear stage after undergoing ovarian stimulation and egg retrieval.

**Table 2:** Reproductive and oncologic outcomes.

Outcome	Total (n = 216)	HUP (n = 39)	CHOP (n = 177)
Reproductive outcomes			
Additional fertility procedure	36 (16.6%)	15 (38.5%)	21 (11.9%)
Oocyte vitrification	35 (16.2%)	14 (35.9%)	21 (11.9%)
Via ovarian stimulation	9 (4.2%)	7 (17.9%)	2 (1.1%)
No. of oocytes retrieved	5 (IQR 4-8)	6 (IQR 5-10)	3 (IQR 2-4)
Patients with MII oocytes	9 (100%)	7 (100%)	2 (100%)
Via ovarian tissue	26 (12.0%)	7 (17.9%)	19 (10.7%)
No. of oocytes retrieved	2 (IQR 1-4)	4 (IQR 2-8)	1 (IQR 1-3)
Patients with MII oocytes	8 (30.8%)	1 (14.3%)	7 (36.8%)
Embryo vitrification	1 (0.5%)	1 (2.6%)	0 (0%)
Follow-up with REI	53 (24.5%)	22 (56.4%)	31 (17.5%)
OTT	1 (0.5%)	1 (2.6%)	0 (0%)
Pregnancy	9 (4.2%)	6 (15.4%)	2 (1.1%)
Unassisted	5 (2.3%)	4 (10.3%)	1 (0.6%)
Assisted	4 (1.9%)	3 (7.7%)	1 (0.6%)
Intrauterine insemination	2 (0.9%)	1 (2.6%)	1 (0.6%)
Embryo transfer	2 (0.9%)	2 (5.1%)	0 (0%)
Live birth	9 (4.2%)	7 (17.9%)	2 (1.1%)
Oncologic treatment and outcomes			
Pre-OTC chemotherapy	146 (67.6%)	22 (56.4%)	124 (70.1%)
Pre-OTC alkylating agent	91 (62.3%)	11 (50.0%)	80 (64.5%)
Post-OTC chemotherapy	208 (96.7%)	33 (84.6%)	175 (98.9%)
Post-OTC alkylating agent	198 (95.2%)	27 (81.8%)	171 (97.7%)
Post-OTC stem cell transplant	154 (71.6%)	15 (38.5%)	139 (78.5%)
Post-OTC radiation	75 (34.9%)	16 (41.0%)	59 (33.3%)
Total body	(64.0%)	5 (31.3%)	43 (72.9%)
Pelvic	(26.7%)	10 (62.5%)	10 (16.9%)
Whole abdomen	(8.0%)	0 (0%)	6 (10.2%)
Craniospinal	(4.0%)	1 (6.3%)	2 (3.4%)
Post-OTC meta-iodobenzylguanidine exposure	7 (3.2%)	0 (0%)	7 (4.0%)
Death	29 (13.4%)	4 (10.2%)	25 (14.1%)

*Note:* Data are means ± standard deviations, medians (IQRs), or n (%). MII = metaphase II; IQR = interquartile range; OTC = ovarian tissue cryopreservation; OTT = ovarian tissue transplantation; REI = reproductive endocrinology and infertility.

**Table 3:** Characteristics of ovarian tissue cryopreservation patients who also underwent ovarian stimulation and retrieval.

Patient	Diagnosis	Age at stimulation (y)	Prior chemotherapy	AMH level (ng/mL) and/or AFC	Oocyte yield
1	Hodgkin lymphoma	20	Yes	—	Stimulation at OSH, 2 MII oocytes frozen
2	Beta thalassemia	20	Yes	AMH, 0.4 AFC, 8	6 oocytes, 4 MII oocytes
3	Chronic myeloid leukemia	26	No	AFC, 13	10 oocytes, 6 MII oocytes
4	Hodgkin lymphoma	22	Yes	—	Stimulation at OSH, 5 MII oocytes frozen
5	Rectal cancer	25	No	AMH, 2.37 AFC, 12	16 oocytes, 13 MII oocytes
6	Invasive ductal breast cancer	29	No	AMH, 4.0	12 oocytes, 8 MII oocytes
7	Cervical cancer	33	No	AMH, 1.2	4 oocytes, 4 MII oocytes
8	Hodgkin lymphoma	22	Yes	AMH, 0.3 AFC, 9	4 oocytes, 1 MII oocytes
9	Acute myeloid leukemia	15	Yes	—	11 oocytes, 8 MII oocytes

*Note:* AMH = antimüllerian hormone; AFC = antral follicle count; MII = metaphase II.

Separately, and distinct from ovarian stimulation with oocyte retrieval, 26 patients cryopreserved oocytes isolated from the ovarian tissue excised during OTC as an adjunct to tissue freezing. Seven HUP patients froze oocytes from ovarian tissue (median, 4 [IQR, 2–8]; 14.3% with MII oocytes), and 19 CHOP patients froze oocytes from ovarian tissue (median, 1 [IQR, 1–3]). Among these 26 patients who froze oocytes from the tissue, 18 underwent OTC before 2020, when all oocytes were vitrified on the day of tissue collection. Fifteen patients froze GVs, one froze an MI, and two froze GVs and MII oocytes. After 2020, six patients had GVs that matured to MII oocytes, one patient had an MI oocyte that progressed to MII, and one patient had MII oocytes frozen without maturation.

At the time of analysis, the mean age of living patients was 19.4 ± 10.9 years (34.1 ± 11.0 years among HUP patients and 16.0 ± 7.5 years among CHOP patients). Of those with follow-up data, at ≥11 years of age and at ≥12 months after procedure (n = 115), 68.8% were diagnosed with POI, and 63.6% initiated HRT. Of the 74 patients (64.3%) who reported initiation or resumption of menses after OTC, half (n = 34, 48.6%) were on HRT, and the rest (n = 38, 51.4%) had spontaneous menses. Among patients with available follow-up data, there was no statistically significant difference in the incidence of POI between those who underwent ovarian biopsy (67/98, 68%) and those who underwent oophorectomy (8/11, 73%) (*P*=1.00). Similarly, resumption of menses did not significantly differ between the biopsy group (69/104, 66%) and the oophorectomy group (5/11, 45%) (*P*=.30). When comparing patients treated at HUP with those treated at CHOP, similar rates of POI incidence (59/82 [72%] at HUP vs. 16/27 [59%] at CHOP, *P*=.32) and resumption of menses (56/87 [64%] at HUP vs. 18/28 [64%] at CHOP, *P*=1.00) were observed. Of note, patients who received chemotherapy before OTC had a significantly higher incidence of POI than those who had not received chemotherapy (56/72 [78%] vs. 19/37 [51%], *P*=.009). This is likely because half of the patients who received chemotherapy before OTC (36/72, 50.0%) underwent stem cell transplant for leukemia before the procedure. However, the rate of menstrual resumption did not differ significantly between the two groups (49/75 [65%] in the pre-OTC chemotherapy group vs. 25/40 [63%] in the no chemotherapy group, *P*=.92). Although only 24.5% (n = 53) of patients in our cohort have had formal follow-up with the reproductive endocrinology and infertility (REI) team to discuss HRT or reproductive planning, most living patients (62.6%) are still <21 years old and likely not ready for family-building.

Only one patient in our cohort underwent autologous OTT. She was a 35-year-old partnered nulliparous woman who was diagnosed with large B cell lymphoma after workup for worsening shortness of breath revealed a 10-cm anterior mediastinal mass. She was hospitalized and advised to begin chemotherapy immediately with cyclophosphamide, doxorubicin, vincristine, prednisone (R-CHOP), and rituximab. An urgent REI consultation was performed in the hospital to discuss fertility preservation. Given the severity of her respiratory symptoms, ovarian stimulation was not feasible. Instead, she elected to undergo OTC via ovarian biopsy after one chemotherapy cycle. An AMH was not completed before chemotherapy and was only completed after chemotherapy (AMH level, 0.3 ng/mL) after serum was banked as part of a research study and, thus, was not clinically available. She was counseled that after chemotherapy, AMH levels are known to decrease because of follicular destruction and are difficult to interpret because they do not reflect the overall ovarian reserve. These levels can rebound months after completion of chemotherapy (10, 11).

The decision to proceed with ovarian tissue biopsy was made on the basis of her planned treatment regimen, which was not felt to be sterilizing. A laparoscopic ovarian biopsy was performed to remove approximately one third of the left ovarian cortex without complications, followed by completion of six cycles of R-CHOP and mantle radiation. After therapy, her menses did not resume, and laboratory evaluation confirmed POI (AMH level, <0.08 ng/mL; follicle-stimulating hormone level, >80 mIU/mL; estradiol, <20 pg/mL). She was started on HRT to manage menopausal symptoms and optimize bone health.

Three years after her diagnosis, she remained in good health with no evidence of disease and reconsulted with REI to discuss options for family-building. Desiring a genetically related child, the patient opted to undergo OTT. A multidisciplinary review was conducted with the patient’s oncologist and a maternal–fetal medicine specialist, who agreed that it was safe for her to attempt pregnancy with OTT. Laparoscopy was performed, and all the frozen ovarian cortical tissue was thawed and sutured to the existing ovaries. Hysteroscopy and chromopertubation of the fallopian tubes were performed at the time of surgery to confirm a normal uterine cavity and tubal patency.

After surgery, she was monitored with serial ultrasound examinations and bloodwork to assess ovarian activity. Six months after transplant, she reported improvement in her menopausal symptoms and was noted to have a dominant follicle on ultrasound. An intrauterine insemination (IUI) was performed; however, she failed to conceive. Menstrual cycles stopped thereafter. She subsequently pursued pregnancy using donor eggs and has delivered two healthy children.

Supplemental Table 1 shows characteristics of the eight patients in our cohort with documented pregnancies and live births after OTC. Pregnancies occurred at a mean of 65 ± 51 months after OTC, and live births were a mean of 81 ± 46 months after OTC. Six of the eight patients who achieved pregnancy received alkylating chemotherapy (mean total cyclophosphamide equivalent dose, 16.1 ± 15.3 G/m^2^). Two patients received stem cell transplant conditioning, both of whom went on to have spontaneous pregnancies. Two of the patients received radiation therapy after OTC—one craniospinal and one pelvic. Five patients who did not undergo OTT had unassisted pregnancies and live births, two patients had live births after IUI, and, as previously mentioned, the OTT patient achieved pregnancies and live births via embryo transfer with donor oocytes. One patient with neuroblastoma who underwent OTC at the age of 28 years via unilateral ovarian biopsy later attempted pregnancy using immature oocytes retrieved from ovarian tissue and vitrified separately. However, none of the oocytes (two GVs and two MI oocytes) survived the thaw.

Among the 208 patients (96.7%) who received chemotherapy after OTC, 198 (95.2%) were exposed to alkylating agents, and 154 (71.6%) underwent stem cell transplant conditioning. There were 75 patients (34.9%) who received post-OTC radiation with potential impact to the ovaries (total body, 64.0%; pelvic, 26.7%; whole abdomen, 8.0%; craniospinal, 4.0%). Seven CHOP patients (4.0%) had post-OTC exposure to meta-iodobenzylguanidine, a systemically administered radioactive material attached to a cell-targeting molecule used in the treatment of high-risk neuroblastoma. To our knowledge, no patients experienced a delay in initiation of cancer therapy because of OTC. Since the procedure, 29 patients (13.4%, 4 from HUP and 25 from CHOP) have died.

## Discussion

Per the American Society for Reproductive Medicine guidelines on fertility preservation, OTC is no longer considered an experimental procedure (1). The benefits of OTC include minimal treatment delay and the ability to preserve autologous fertility potential, particularly in premenarchal patients. Additionally, OTT can provide the opportunity for pregnancy, although some patients conceive unassisted.

A long-standing collaborative program between CHOP and HUP was established in 2008 to offer OTC to patients at risk of POI. Initially, OTC was offered as part of a research protocol at both institutions; however, at HUP, OTC is now available through a clinical protocol, allowing more patients to undergo the procedure on the basis of provider discretion. Although most patients (96.7%) in our cohort received chemotherapy after OTC, with 95.2% exposed to alkylating agents, there were four patients at CHOP who were eligible for and underwent OTC on the basis of planned treatment but ultimately did not receive any gonadotoxic agents.

Slow freeze and vitrification techniques for cryopreserving ovarian tissue appear to be equivalent, with studies showing no differences in follicular morphology and distribution and reassuring clinical outcomes after OTT using vitrified ovarian tissue, including two documented live births (12, 13). All procedures at our institution were performed using the slow freeze technique. However, vitrification could potentially expand patient access to OTC because it circumvents the need for the costly equipment, specialized training, and logistical coordination required for slow freeze.

Although OTC is the only fertility preservation method available to premenarchal patients, postmenarchal patients also have the option of ovarian stimulation and cryopreservation of oocytes and embryos. As a result, we see higher utilization of OTC among pediatric patients, whereas adult patients often opt for ovarian stimulation with subsequent oocyte/embryo cryopreservation because these are more established methods. Studies have shown that in healthy women aged <35 years, cryopreserving 10 mature oocytes provides a cumulative live birth rate of approximately 60.5%; however, live birth rate may decrease as low as 34% in oncofertility patients, possibly because of inferior oocyte quality in patients with cancer(4, 14). On the other hand, OTT in young women yields live birth rates per transplantation of 25%–36% (4). One study comparing OTC and oocyte vitrification in patients undergoing gonadotoxic treatment demonstrated a trend toward higher live birth rate in patients who underwent oocyte vitrification; however, almost half of patients who underwent OTT in their cohort had spontaneous pregnancies (15).

In our cohort, nine patients (4.2%), mostly from the HUP group, underwent both ovarian stimulation and OTC. A recent review article analyzing outcomes data of these two methods concluded that combining oocyte vitrification and cryopreservation of ovarian tissue in patients with cancer could yield a live birth rate closer to 50%–60% (4). Oocyte cryopreservation is considered an established procedure with higher success rates than OTC and is, therefore, typically attempted first. In this series, some patients had leukemia, historically thought to be a high-risk condition for subsequent tissue transplantation, so they underwent an attempt at oocyte cryopreservation, even after exposure to chemotherapy. Other patients who yielded few oocytes or had concerns about oocyte quality because of recent chemotherapy opted for OTC as an additional fertility preservation strategy. Although some have advocated for OTC to be performed first, followed by oocyte cryopreservation, to avoid operating on an enlarged ovary with multiple corpora lutea, it is possible that oocyte yield may be lower in patients who have had a large portion of the ovary removed. Nevertheless, for patients undergoing stimulation and subsequent OTC, it is generally recommended to delay OTC for at least two weeks after stimulation to allow for resolution of ovarian edema and to facilitate safer tissue handling and cryopreservation. However, this timeline is not always feasible in urgent fertility preservation settings. Because of significant time constraints owing to impending chemotherapy, four patients in our cohort underwent OTC at <2 weeks after oocyte stimulation and retrieval. Patients who had a suboptimal oocyte yield from stimulation in our cohort either had previous chemotherapy exposure or had documented diminished ovarian reserve before stimulation.

Ovarian tissue cryopreservation and OTT procedures are safe procedures with low operative complication rates. Laparoscopy has been shown to be a safe and feasible option for the surgical treatment of benign ovarian disease in the pediatric population, associated with less blood loss and shorter operative times than open surgery (16, 17). Previous studies specifically analyzing OTC/OTT postoperative complications have cited complication rates as low as 0.2% after OTC and 1.4% after OTT (18). In our cohort, all procedures were performed laparoscopically with no conversions to laparotomy, and only two patients (0.9%) had documented procedure-related complications.

Nevertheless, among the patients who experienced complications, one had significant bleeding after biopsy, highlighting the potential risk of biopsy compared with oophorectomy. For this reason, patients with a high risk of ovarian failure should be counseled about these risks and possibly recommended for oophorectomy over biopsy. However, our CHOP protocol remains conservative in terms of who is offered oophorectomy, and most pediatric patients, such as this patient who was seven years old at OTC, are counseled toward biopsy over oophorectomy given the limited live births from OTC in pediatric survivors and the fact that some girls regain ovarian function and achieve pregnancy even after high-risk treatment. The delayed presentation and ultimate diagnosis of this postoperative bleed was atypical, and it was likely attributable to a slow venous bleed from the biopsy site that may have self-resolved if the patient had not been so thrombocytopenic from her high-dose chemotherapy. Our pediatric surgeon also recommends considering oversewing the ovary after biopsy, rather than just relying on electrocautery, to help prevent bleeding complications in patients with impending treatment. Although there is no formal guideline or high-level evidence specifically addressing the optimal interval between OTC surgery and the initiation of chemotherapy, patients should be counseled on specific postoperative risks related to their subsequent treatment, especially in cases where chemotherapy cannot be delayed.

One concern for patients with cancer considering OTC is the potential for reintroduction of malignant cells during OTT, which could lead to cancer recurrence. Cancers including breast, colorectal, stomach, appendix, and gynecologic primary cancers (including endometrial and cervical cancer) are most likely to metastasize to the ovary; therefore, cryopreserved tissue in patients with these malignancies may carry a higher risk of reseeding tumor cells after transplantation (19). Leukemia, a systemic cancer in nature, is thought to have the highest risk of reintroducing malignant cells after transplant, approaching 11% (20, 21). Inducing remission with chemotherapy before OTC, resulting in bone marrow without detectable disease, reduces the risk of malignant cells in the harvested ovarian tissue but may also compromise ovarian reserve (11). However, several live births have been documented after OTT in patients with leukemia who underwent OTC after achieving complete remission, suggesting that cumulative alkylating chemotherapy dose before OTC may not significantly impact the follicular pool, especially in younger patients with high ovarian reserve at the time of the exposure (6, 22).

For patients with high-risk disease, testing including histologic, immunohistochemical, and molecular screening of ovarian tissue, as well as murine xenotransplantation of ovarian tissue to screen for disease recurrence before autologous transplant, is performed at some centers to minimize the risk of reintroducing malignant cells and increase OTT safety (23). Reassuringly, in a recent case series of six patients with leukemia who underwent OTC after established remission but before stem cell transplantation and subsequent OTT, all patients remained relapse-free after a median follow-up of 51 months (24). In our cohort, all 69 patients with leukemia who underwent OTC were in remission at the time of OTC in preparation for bone marrow transplant. The only patient in our cohort who underwent OTT had a diffuse large B cell lymphoma, a malignancy considered a lower risk of this complication.

Another consideration for patients undergoing OTC is cost. In Pennsylvania, there is no state mandate for insurance coverage of fertility preservation, and OTC/OTT is not reliably covered by private insurance. At HUP, surgical costs are typically covered for patients with commercial insurance; however, patients should pay for the tissue processing and cryopreservation. At CHOP, all funding for pediatric OTC procedures is provided through research grants and philanthropic funds. Storage of the cryopreserved tissue presents another cost consideration and potential financial burden. Additionally, there is currently no funding available at our institution to offset the costs of the OTT procedure, creating an additional barrier to use of the tissue in those without coverage. One limitation of this study is the lack of data on patients who may have been eligible for OTC but did not undergo the procedure, whether because of financial barriers, personal choice, or other access-related factors; therefore, using our current data set, we are unable to assess how cost may have influenced procedure patterns or tissue utilization.

Unsurprisingly, over two thirds (68.8%) of patients with follow-up data were ultimately diagnosed with POI after OTC. Although prior studies of OTC patients have documented higher rates of POI of 77%–97%, our lower rate may be attributed to the high rate of loss of follow-up (49.5%) in our cohort (6). To manage the sequelae of chronic hypoestrogenism, 63.6% of patients with follow-up data were prescribed HRT. Although the primary and currently accepted indication for OTT is to restore fertility and enable future pregnancy, a potential added benefit is the restoration of endogenous hormone production, which may help alleviate menopausal symptoms in some patients. Studies have shown that 95% of patients demonstrate endocrine function, evidenced by return of menses, after OTT; however, graft effects may be limited (6, 25). Recovery of ovarian function after OTT takes 60–240 days and can last up to 7 years (26).

Of note, among those in our cohort who underwent OTC but not OTT, five patients had unassisted pregnancies with live births, and two patients had live births after IUI. Although the literature does not comment on likelihood of pregnancy and live birth rates after OTC without OTT, studies estimate the natural conception rate of 40% among OTC patients after OTT (27). In our cohort, all five patients with unassisted pregnancies and live births were ≥20 years of age at the time of OTC, four of the five were exposed to alkylating chemotherapeutics, and two underwent highly gonadotoxic stem cell conditioning regimens. Both older age at the time of exposure and the increased gonadotoxicity of these chemotherapy regimens are known risk factors for subsequent POI; however, these patients demonstrate that predictions on fertility are not always reliable, and some patients may still conceive naturally after treatment. Therefore, we continue to recommend OTC broadly to patients at risk of gonadotoxicity, recognizing that even those receiving high-risk treatments may retain fertility potential.

**Figure 1:**
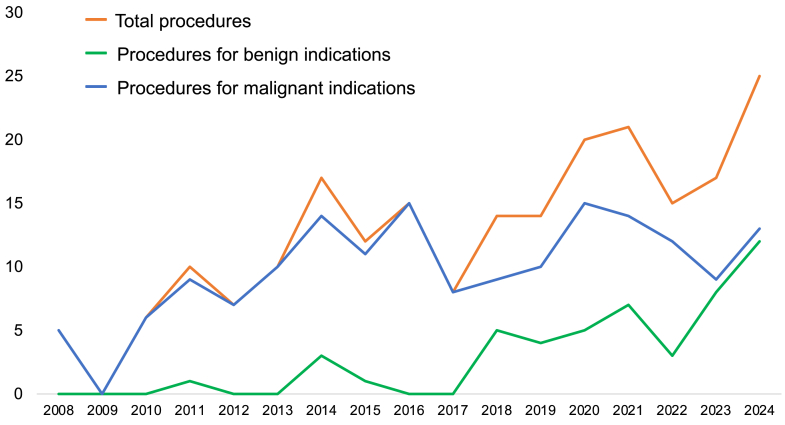
Trends in ovarian tissue cryopreservation procedures performed at Hospital of the University of Pennsylvania and at Children’s Hospital of Philadelphia since 2008, shown as total procedures (orange line), procedures performed for benign indications (green line), and procedures performed for malignant indications (blue line).

Our center has seen a growing trend in OTC procedures over time. As shown in Figure 1, the number of OTC procedures performed has increased since our program’s initiation in 2008. Although tissue utilization rates are very low, OTC can provide patients with hope, often during a time of emotional and psychological stress as they face a new diagnosis or prepare for intensive treatment.

## Conclusion

At our collaborative program between HUP and CHOP, over 200 patients have undergone OTC. OTC offers both premenarchal and postmenarchal patients the potential to preserve autologous fertility and restore some ovarian function later in life. Ovarian tissue cryopreservation can provide the opportunity for pregnancy, although some patients conceive without assistance. At our institution, only one patient has returned for OTT, highlighting the importance of continued tracking of long-term outcomes and patient engagement after OTC. Additional studies are needed to understand the factors that influence tissue utilization to improve counseling and increase transplantation rates.

## CRediT Authorship Contribution Statement

**Meridith P. Pollie:** Writing – review & editing, Writing – original draft, Visualization, Validation, Software, Project administration, Methodology, Investigation, Formal analysis, Data curation, Conceptualization. **Caitlin E. Martin:** Writing – review & editing, Writing – original draft, Supervision, Methodology, Data curation, Conceptualization. **Margaret A. Rush:** Methodology, Data curation, Conceptualization. **Claire Carlson:** Writing – review & editing, Validation, Data curation. **Jeanne Ricci:** Data curation. **Maddie Trego:** Data curation. **Peter Mattei:** Writing – review & editing. **Suneeta Senapati:** Writing – review & editing, Supervision, Methodology, Investigation, Conceptualization. **Jill P. Ginsberg:** Writing – review & editing, Writing – original draft, Visualization, Supervision, Methodology, Investigation, Data curation, Conceptualization. **Clarisa R. Gracia:** Writing – review & editing, Writing – original draft, Visualization, Validation, Supervision, Resources, Project administration, Methodology, Investigation, Data curation, Conceptualization.

## Declaration of Interests

M.P.P. has nothing to disclose. C.E.M. has nothing to disclose. M.A.R. has nothing to disclose. C.C. has nothing to disclose. J.R. has nothing to disclose. M.T. has nothing to disclose. P.M. has nothing to disclose. S.S. reports funding from the National Institutes of Health, AbbVie, Burroughs Wellcome Fund, and the American Academy of Sleep Medicine outside the submitted work and the Society for Assisted Reproductive Technologies Executive Council. J.P.G. has nothing to disclose. C.R.G. reports American Society for Reproductive Medicine Practice Committee Chair and Chair, Reproductive Endocrinology and Infertility Division, American Board of Obstetrics and Gynecology.
