# Supplementary material for: Characterizing patients who underwent ovarian tissue cryopreservation at a large academic center in the United States
Source: F S Rep. 2025 Oct 1;6(4):487–96. doi: 10.1016/j.xfre.2025.09.006 (PMC12746891; doi:10.1016/j.xfre.2025.09.006)
Supplement: Supplemental Figure 1 [file mmc1.pdf]

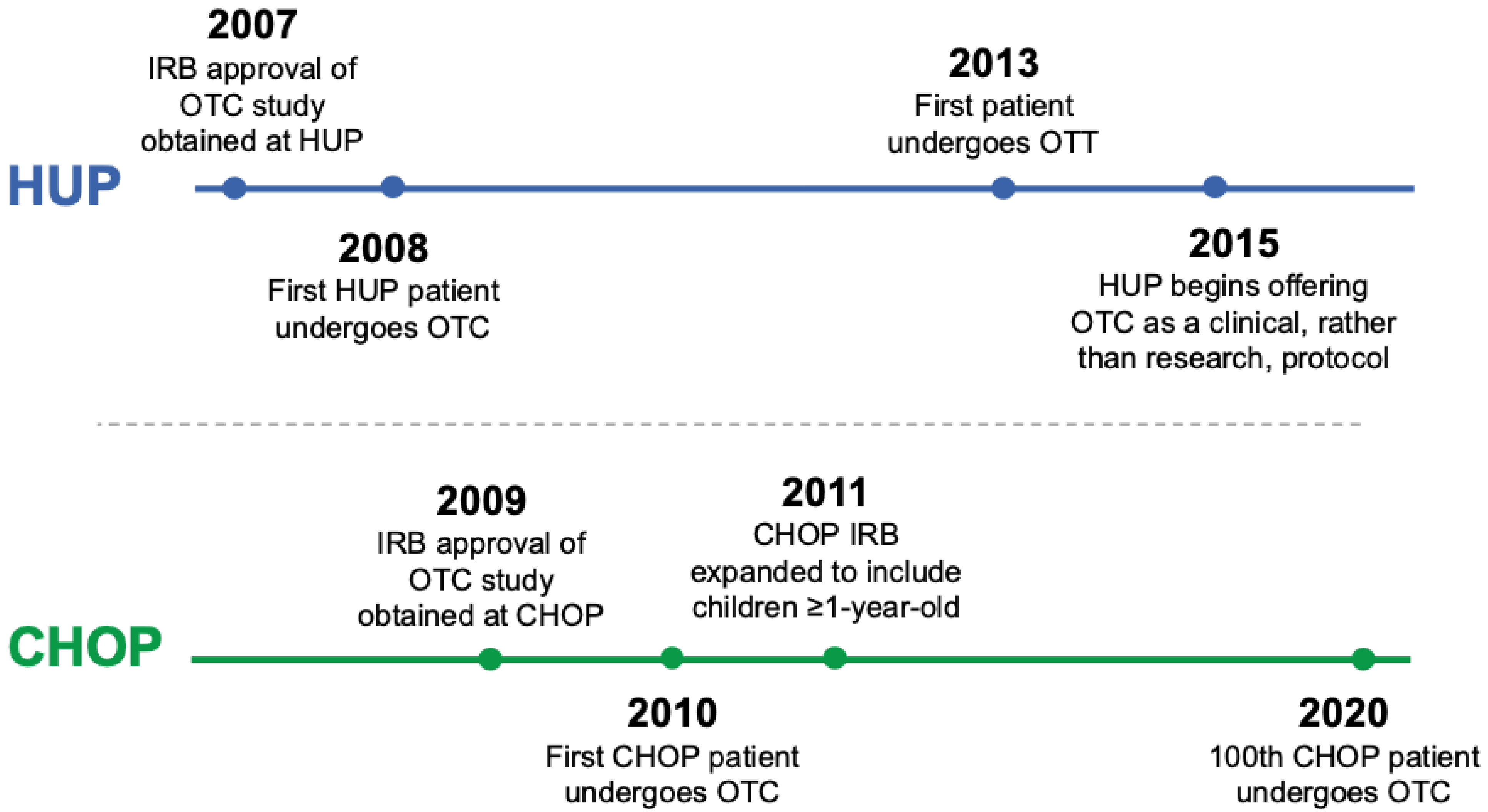

Supplemental Figure 1. Timeline of key institutional and clinical milestones related to OTC at HUP (blue line) and CHOP (green line).
