# Supplementary material for: Characterizing patients who underwent ovarian tissue cryopreservation at a large academic center in the United States
Source: F S Rep. 2025 Oct 1;6(4):487–96. doi: 10.1016/j.xfre.2025.09.006 (PMC12746891; doi:10.1016/j.xfre.2025.09.006)
Supplement: Supplemental Table 1 [file mmc2.docx]

Supplemental Table 1. Characteristics of OTC patients with live births.

| **Patient** | **Diagnosis** | **Age at OTC** | **OTC type** | **Pre-OTC exposure** | **Post-OTC exposure** | **Pregnancy details** | **Live birth** |
| --- | --- | --- | --- | --- | --- | --- | --- |
| 1 | Large B-cell lymphoma | 35 | Unilateral biopsy | Alkylating chemotherapy | Alkylating chemotherapy | 1) OTT;  2) IUI;  3) IVF with donor oocytes | 1) No;  2) No;  3) Yes |
| 2 | Hodgkin lymphoma | 20 | Unilateral biopsy | Alkylating chemotherapy | Alkylating chemotherapy, bone marrow transplant conditioning | Unassisted | Yes |
| 3 | Hodgkin lymphoma | 33 | Unilateral biopsy | Non-alkylating chemotherapy (ABVD) | Pelvic radiation | Unassisted | Yes |
| 4 | B-cell lymphoma | 31 | Unilateral biopsy | Alkylating chemotherapy | Alkylating chemotherapy | Unassisted | Yes |
| 5 | Hodgkin lymphoma | 29 | Unilateral oophorectomy | Non-alkylating chemotherapy (ABVD) | Alkylating chemotherapy, bone marrow transplant conditioning | Unassisted | Yes |
| 6 | Invasive ductal breast cancer | 29 | Unilateral biopsy | None | Alkylating chemotherapy | Unassisted | Yes |
| 7 | Pituitary germinoma | 19 | Unilateral biopsy | None | Craniospinal radiation | IUI | Yes |
| 8 | Ewing sarcoma | 16 | Unilateral biopsy | None | Alkylating chemotherapy | 1) IUI;  2) Unassisted | 1) Yes;  2) Yes |

OTC, ovarian tissue cryopreservation; OTT, ovarian tissue transplantation; IUI, intrauterine insemination; IVF, in vitro fertilization; ABVD, adriamycin (doxorubicin), bleomycin, vinblastine, and dacarbazine.
